# Refinement of a Parent–Child Shared Asthma Management Mobile Health App: Human-Centered Design Study

**DOI:** 10.2196/34117

**Published:** 2022-02-17

**Authors:** Jennifer Sonney, Emily E Cho, Qiming Zheng, Julie A Kientz

**Affiliations:** 1 Department of Child, Family, and Population Health Nursing School of Nursing University of Washington Seattle, WA United States; 2 Department of Human Centered Design and Engineering University of Washington Seattle, WA United States

**Keywords:** parent–child shared management, school-age children, asthma, participatory design, mHealth, prototype, usability, family health informatics

## Abstract

**Background:**

The school-age years, approximately ages 7 through 11, represent a natural transition when children begin assuming some responsibility for their asthma management. Previously, we designed a theoretically derived, tailored parent–child shared asthma management mobile health app prototype, Improving Asthma Care Together (IMPACT).

**Objective:**

The purpose of this study was to use human-centered design (HCD) to iteratively refine IMPACT to optimize user experience and incorporate evidence-based longitudinal engagement strategies.

**Methods:**

This study used a mixed methods design from December 2019 to April 2021. Our app refinement used the HCD process of research, ideation, design, evaluation, and implementation, including 6 cycles of design and evaluation. The design and evaluation cycles focused on core app functionality, child engagement, and overall refinement. Evaluation with parent–child dyads entailed in-person and remote concept testing and usability testing sessions, after which rapid cycle thematic analyses identified key insights that informed future design refinement.

**Results:**

Twelve parent–child dyads enrolled in at least one round of this study. Eight of the 12 child participants were male with a mean age of 9.9 (SD 1.6) years and all parent participants were female. Throughout evaluation cycles, dyads selected preferred app layouts, gamification concepts, and overall features with a final design prototype emerging for full-scale development and implementation.

**Conclusions:**

A theoretically derived, evidence-based shared asthma management app was co-designed with end users to address real-world pain points and priorities. An 8-week pilot study testing app feasibility, acceptability, and preliminary efficacy is forthcoming.

## Introduction

Over 5 million children in the United States have asthma, making it the most common chronic condition of childhood [1]. Asthma management is largely dependent upon symptom recognition, monitoring, and response as well as timely and appropriate medication use [2,3]. Despite national asthma management guidelines, it is estimated that over 50% of US children with asthma are uncontrolled, placing them at higher risk of exacerbation and poorer outcomes [4]. Childhood asthma exacerbations account for 767,000 emergency department visits, 74,000 hospitalizations, and 13.8 million missed school days annually [1]. As a consequence, children with poor asthma control and their parents experience lower quality of life and negative academic and work performance [5-7].

The school-age years, approximately ages 7 through 11, represent a natural transition in asthma management responsibility. School-age children must start assuming some responsibility for asthma-related care as they spend increasing time away from parents at school and other extracurricular activities [8]. Unfortunately, there is frequent disagreement between parents and children with respect to asthma symptom frequency and severity, asthma management practices, and overall level of control, with parents often reporting fewer symptoms, higher medication adherence, and better control than their children [9-12]. This disagreement may be contributing to an overestimation of childhood asthma control and subsequent undertreatment. Careful and deliberate parent–child shared asthma responsibility improves asthma symptom assessment, medication adherence, and overall asthma control [13-15]. A critical gap among existing asthma management interventions is the failure to account for and facilitate shared management responsibility. A parent–child shared asthma management solution is needed to facilitate optimal comanagement of asthma and to prepare the child to assume increasing asthma management responsibility.

With an estimated 85% of US adults owning a smartphone [16] and 69% tracking their health online [17], the ubiquity of smartphones has led to an explosion of mobile health (mHealth) self-management apps. Among adults with asthma, effective mHealth interventions combine medical guidelines, personalized self-monitoring, and behavior change techniques [18-20]. Presently, there are far fewer mHealth asthma apps for youth, with the majority specifically designed for adolescents [21-23]. While many of these apps have demonstrated preliminary efficacy [23], they are designed to support self-management versus parent–child shared asthma management, which limits their utility in school-age children. Among the apps specifically developed for children, they are almost entirely focused on education rather than engaging the child in assuming some responsibility for monitoring their own health [21]. While school-age children are often familiar with using a smartphone, only an estimated 17% of US children have their own smartphone [24], therefore an app designed for a parent and child to use together to support asthma management represents an important area of opportunity. To that end, there is now a need for mHealth interventions that leverage lessons learned from the adult literature and are specifically designed to facilitate parent–child shared management of asthma.

Human-centered design (HCD) is an approach to participatory design wherein end users are engaged throughout the iterative design process [25,26]. Previously, we reported on our use of HCD to develop a preliminary prototype of a parent–child shared asthma management mHealth intervention and companion wearable device [27]. The prototype features and functions were developed to address asthma management needs and priorities identified by parent–child dyads. These app features and functions were also evaluated and approved by an asthma clinician to ensure they aligned with the national asthma management guidelines [2,3]. Likewise, the prototype was theoretically derived from Social Cognitive Theory and the Common Sense Model of Parent–Child Shared Regulation [28,29]. Social Cognitive Theory stresses that goal setting, action planning, and self-monitoring are important behavior change processes. The Common Sense Model of Parent–Child Shared Regulation emphasizes the contributions that both parent and child make toward shared management of health. Therefore, the app was specifically designed for parent–child dyads to use together to facilitate shared management; each week, parents and children select a small, achievable shared management goal, review goal-specific guidance, anticipate barriers, and monitor their goal progress on the subsequent week. Notably, this original prototype prioritized the integration of behavior change, personalized self-monitoring, and medical guidelines [27,30].

The true viability of any mHealth behavior change intervention app is dependent upon ongoing use. While our study team iteratively developed the features and functions of the original app prototype, in-depth usability testing of user experience (UX) and subsequent design iteration were beyond the scope of the original project. Refinement was necessary to ensure that the UX was optimized. Similarly, the literature has clearly shown that overall mHealth app engagement wanes over time, thus limiting the potential efficacy of any app [31]. Given that this mHealth app was designed to function as a behavioral health intervention, integration of engagement strategies was also necessary to promote longitudinal engagement with app. Therefore, before proceeding to full-scale app development and pilot testing, the purpose of this study was to use HCD to refine the original parent–child shared asthma management mHealth prototype, Improving Asthma Care Together (IMPACT), to optimize UX and engagement. The specific aims were to (1) assess and iteratively refine the mHealth app based upon usability findings, (2) incorporate longitudinal engagement strategies within the app, and (3) innovate a home-based multimodal solution to overcome barriers imposed by the COVID-19 pandemic.

## Methods

### Design and Sample

This study used a mixed methods design and included a series of design-evaluation cycles from December 2019 to April 2021. Study recruitment occurred from December 2019 to December 2020. A convenience sample of fourteen 7- to 11-year-old children with asthma and one of their parents was recruited from the principal investigator’s (JS) research database, which includes parent–child dyads who have participated in previous studies for school-age children with asthma. Recruitment for these prior studies included flyer distribution by school nurses as well as study flyer posting in pediatric provider offices, community locations (eg, libraries, Boys and Girls clubs), and social media. Child eligibility included (1) age 7-11 years, (2) parent-reported asthma diagnosis, (3) parent-reported prescription for daily asthma medication, and (4) able to speak and understand English. The prescription for daily asthma medication was used as a proxy to indicate persistent asthma, considered more severe than intermittent or exercise-induced asthma [3]. Parent/caregiver inclusion criteria included (1) 18 years or older, (2) child’s primary caregiver, (3) able to understand and read English, (4) reside with the child for at least 50% of the time, and (5) a legal guardian who may consent for the child to participate. Exclusion criteria included prior participation in the original prototype development as well as current asthma exacerbation, such as wheezing or respiratory distress, as this is a serious health event that requires careful monitoring and would distract dyads from participation. However, dyads were eligible to participate once the exacerbation resolved.

### Ethics

The University of Washington Human Subjects Division reviewed this study and deemed it as exempt (#00003144). Written informed consent was obtained from all parent participants, on behalf of themselves and their child, and assent from all child participants.

### Original IMPACT Prototype

IMPACT was designed to serve as an asthma monitoring system as well as behavioral intervention to promote parent–child shared asthma management. The original IMPACT prototype comprised 3 key features: asthma symptom tracking, asthma control (measured by the Childhood Asthma Control Test or C-ACT [32]), and asthma shared management goal setting and progress reporting. Child-reported asthma symptom events are tracked with the app dashboard in graphical format. Likewise, the app was designed to prompt parent–child dyads to complete the C-ACT weekly, with scores visualized over time in the app dashboard. Finally, each week, parent–child dyads would select 1 or 2 asthma shared management goals that were provided by the app. The following week, dyads would report on their goal progress. Overall, shared management was supported by gradually transferring asthma management responsibility to the child while supported and supervised by the parent. Within the app, the parent–child dyad selects a weekly asthma management task or activity for which the child will begin assuming responsibility. Goal-specific guidance and support (when available) are provided, such as scheduling medication reminders, to assist the dyad with achieving the goal. Such intentional shifting of asthma management responsibility through mutual goal setting ensures the child learns critical management tasks and skills while supported by the parent [9,10,29].

### App Refinement Process

#### Overview

**Figure 1:**
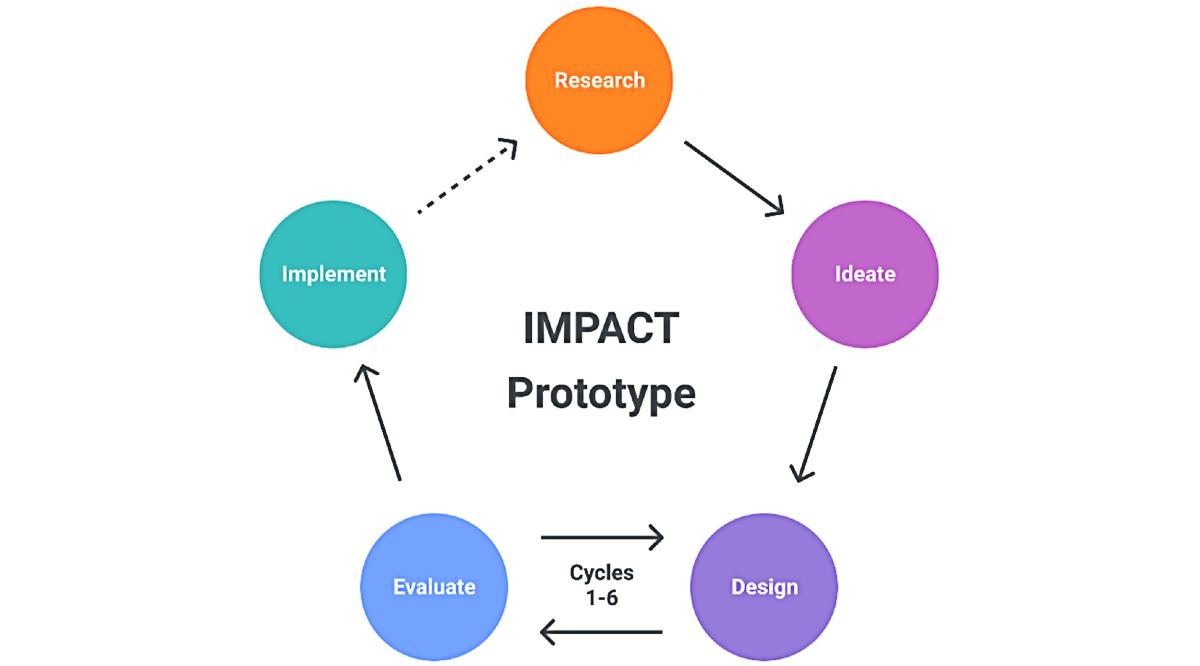
Human Centered Design Process.

A hallmark of HCD is design iteration that follows a cyclic as opposed to linear process (Figure 1). The core study team included a designer (QZ), a pediatric asthma clinician-scientist (JS), a UX researcher (EEC), and a senior HCD expert (JAK). This study followed the HCD process steps: research, ideate, design, evaluate, and implement to refine the IMPACT prototype and incorporate longitudinal engagement strategies [25,33]. The app refinement process required numerous cycles of design and evaluation, which is expected in HCD.

#### Research

During this phase, our team conducted a scoping literature review of mHealth app engagement strategies. Next, our team conducted a market research review to understand existing apps designed for use by school-age children, including health- and non-health–related apps. We then assessed the extent to which existing asthma apps facilitated parent–child shared management.

#### Ideation

**Figure 2:**
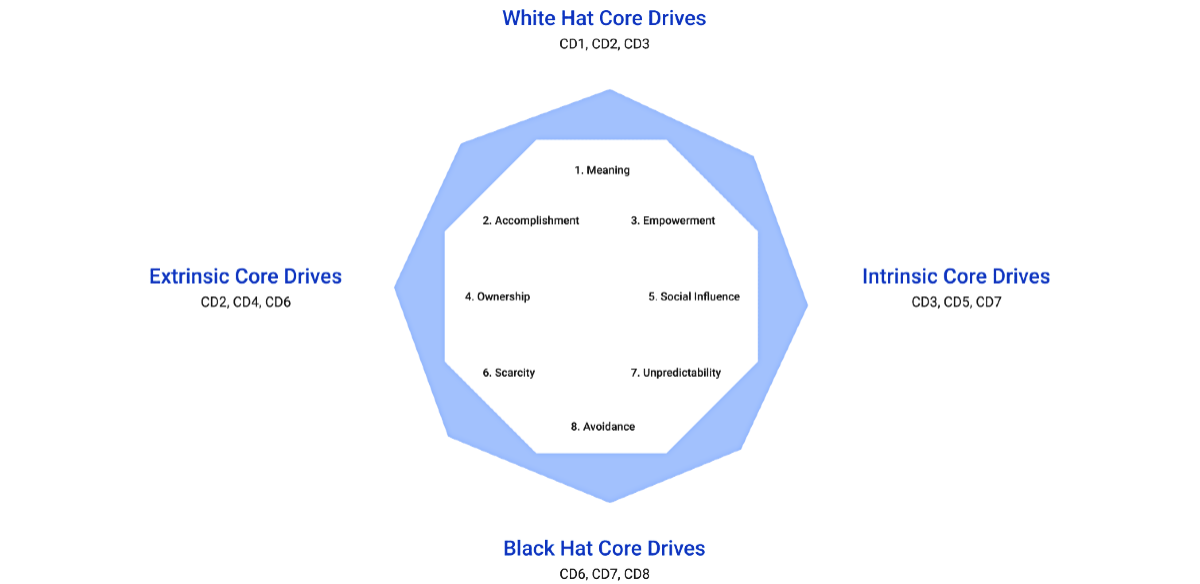
Octalysis framework and core drive motivations. Adapted, with permission, from [38].

The ideation phase of HCD entails generating ideas and solutions to address user needs and priorities [33]. To guide our work in designing for engagement, our team conducted a literature review of common approaches to promoting app engagement [18,24,30,34,35]. Based upon this review, our team decided to incorporate gamification to promote app engagement and subsequently adopted the Octalysis Framework for Gamification and Behavioral Design [36]. Guided by the concept of motivation from social psychology, the Octalysis Framework intentionally accounts for intrinsic, extrinsic, positive, and negative motivations. Intrinsic motivation refers to activities performed out of pure enjoyment with no tangible rewards, whereas extrinsic motivation refers to behaviors performed in pursuit of tangible external rewards [37,38]. Positive motivators stimulate positive emotions such as joy, satisfaction, and a sense of meaning, whereas negative motivators may elicit feelings such as surprise, fear, or concern for a loss of progress or reward. The Octalysis Framework is depicted as an octagon with each side representing 1 of 8 core drives (CDs) of motivation (Figure 2) [36]. CDs associated with extrinsic motivation (CD 2, 4, 6) are located to the left of the model, whereas those associated with intrinsic motivation are represented on the right (CD 3, 5, 7). Positive motivators are located at the top of the model (CD 1, 2, 3), whereas the bottom portion of the model represents negative motivators (CD 6, 7, 8; Figure 2).

Guided by the Octalysis Framework, the study team conducted brainstorming sessions for new features and enhancements of the IMPACT app, using rough sketches and short descriptions to communicate and align ideas across the team. Next, we employed affinity mapping, where ideas were grouped into themes, and team members ultimately voted on their top engagement ideas. Finally, we used effort versus impact matrices to prioritize design ideas and refinements. Although parent–child dyads were not engaged in the initial ideation phase, their feedback from subsequent stages, including new app features or concepts, were prioritized in future design iterations (see the “Results” section for details).

#### Design

The original mHealth IMPACT app prototype was developed using Figma, a digital design and prototyping platform that supports the prototyping spectrum from wireframes through high-fidelity interactive prototypes (Figma, Inc). Given that the original prototype was housed within Figma, the designer (QZ) continued to use Figma for subsequent design ideas to facilitate a cohesive UX. During the design phase, the designer digitally drafted the prioritized design ideas, usually providing 2 or 3 variations. The study team reviewed the designs together and typically selected 2 versions to move forward to user testing in the evaluation phase. The inclusion of the asthma clinician-scientist study team member during such early design stages ensured that prospective designs represented asthma management best practices. As is depicted in Figure 1, numerous rounds of design and evaluation occurred as the app was progressively refined. Initial design rounds used low-fidelity wireframes to depict the basic app structure (information architecture) and functionality (eg, screens showing paths users take, or “user flows”). Later design rounds focused on visual design and microinteractions of the app features, which necessitated high-fidelity prototyping within Figma.

#### Evaluate

The study team used numerous techniques within the evaluation stage, including concept testing, usability testing, and semistructured interviews [33,39,40]. Concept testing entails seeking feedback and preferences from users about specific concepts and designs. For example, presenting different layouts of a dashboard to determine which was more effective for users, a technique known as parallel prototyping [41]. We also used card sorting, which entailed presenting numerous feature options generated from the ideation phase to users and asking them to categorize their preferences in order of importance.

Usability testing, by contrast, was used to test specific tasks within the app to determine whether users were able to use the app as intended [33,39,40]. These tasks are referred to as “flows,” representing the series of screens users encounter as they perform a task. Usability sessions were moderated by the UX researcher (EC) and observed by the designer, which facilitated understanding user feedback. Through the use of the “think-aloud” technique, a cognitive interviewing method, participants were asked to perform a task within the app while the researcher observed [42]. Users were asked to describe their use of the app as they performed the tasks, including what they liked or disliked, what was confusing, and whether they would change anything. All evaluation sessions also incorporated semistructured interviews to elicit any user feedback, suggestions, or other comments.

Following each evaluation session, the UX researcher and designer debriefed the session and discussed their takeaways from the session (eg, user preferences for specific designs, or challenges using a specific flow). As is typical in HCD, each usability testing cycle was followed by rapid thematic analyses to identify key insights [25]. Key insights were then translated into recommended refinements by the UX researcher. These refinements were prioritized using a common usability scoring system (1=highest priority, 4=lowest priority) [43]. Level 1 (high priority) items prevent users from completing a task, Level 2 items create a significant delay in task completion, Level 3 have minor effects on usability, and Level 4 (lowest priority) are subtle with minimal effects. For this project, high-priority items reflected essential functions, new features, or other substantive app changes. Substantive design changes always were tested in a future evaluation cycle. Conversely, lower-priority items—often minor functionality issues such as type of clock face or button—were revised without further testing. Depending on the evaluation findings, the study team decided to either iterate the design further (re-enter the design phase) or move the prototype to implementation. We originally planned 3 cycles of design and evaluation, but as a result of delayed study timelines due to the COVID-19 pandemic, we extended our threshold for concluding iterations to Spring 2021.

#### Implement

Implementation entails handoff of the final designs to the developer team for full-scale development of the app. Implementation usually follows numerous rounds of design and evaluations until a final design emerges.

### Procedures for Evaluation and Refinement

As depicted in Figure 1, this study consisted of research, ideation, numerous design, and evaluation cycles, followed by implementation. Study participants were directly involved in evaluation sessions. Procedures for engagement with study participants during evaluation sessions are herein described. For each session, the UX researcher generated an evaluation session plan including introductory script, task planning (concept testing or usability testing), and open-ended questions for the semistructured interview. Sessions were planned such that child activities were prioritized first to retain their attention.

Once the session plan was complete, the UX researcher contacted the parent of prospective parent–child study participants in the principal investigator’s research database via email. Those who were interested scheduled a study session with the UX researcher. Prior to the COVID-19 pandemic, study sessions were conducted at participant homes or a community library. Participant use of the Figma prototype app was recorded using Mr. Tappy (Mr. Tappy), a kit comprising a magnetic base that attaches to a mobile device with a digital camera on an adjustable metal arm. The UX researcher was able to view the user’s actions via the Mr. Tappy browser-based viewer.

During the COVID-19 pandemic, the stay-at-home orders necessitated to transition to a remote study protocol. All remote study sessions were conducted via Zoom videoconference (Zoom Video Communications, Inc.). As an alternative to Mr. Tappy, which would require mailing to and from participant homes, the UX researcher would access the Figma app prototype, share their screen, and enable “remote control” of their mouse and keyboard. These settings allowed users to freely control their engagement with the app while Zoom recorded the session.

Following informed consent and child assent (electronic consent and assent for remote sessions), the UX researcher moderated the study session following the session plan while the designer observed and took notes. Sessions were approximately 60 minutes in duration. Rapid cycle thematic analyses were completed after each session, as discussed earlier, which informed the subsequent design refinements. Parent–child dyads received US $50 digital gift card following each evaluation session.

## Results

### Participants

Twelve parent–child dyads enrolled in at least one round of this study. Eight of the 12 child participants were male with a mean age of 9.9 (SD 1.6) years. Nine of the child participants identified as White, 2 as Black, and 1 as mixed race. All of the parent participants identified as female, which is consistent with our prior study samples. Nine parent participants identified as White, 2 as Black, and 1 as mixed race. None of the study participants identified as Hispanic or Latinx.

Sample sizes for the 6 evaluation cycles ranged from 3 to 6 dyads. Usability best practices call for 4 or 5 participants per session, which typically will identify 80% of usability problems [44,45]. Larger samples are generally considered overly burdensome, redundant, and time-consuming. Parent–child dyads participated in at least one evaluation session, with 1 dyad participating in 5 sessions (Table 1). The inclusion of some dyads in multiple cycles was beneficial as it ensured they were familiar with the app objectives and core functionality, allowing them to swiftly focus on new design enhancements and changes. By contrast, inclusion of novel dyad users within study cycles, particularly later cycles, ensured we had diverse perspectives and feedback.

**Table 1:** Dyad participation by evaluation cycle (N=12 parent–child dyads).

Dyad number	Evaluation cycle
1	1, 3, 4, 5, 6
2	1, 2, 4, 6
3	1, 3, 4, 5, 6
4	4, 5, 6
5	1
6	4
7	5, 6
8	5
9	2
10	1
11	1, 3
12	2, 4

### Research

#### Engagement Strategies

We found that gamification, or the incorporation of gaming elements in nongaming uses, is increasingly employed in the mHealth market [35,46]. Originating from the videogaming industry, gamification integrates fun elements and human motivation to maintain user engagement. Gamification strategies appear highly variable and dependent upon the user, context, and activity or goal. Some examples include badges, leader boards, social engagement, and challenges or quests [35]. Despite this variability, gamification shares the ultimate goal of motivating users to continue using the app [47].

#### Existing Apps for Children

Among existing apps for children, we found several common strategies, including the use of avatars, badges, and streaks. Similarly, child-facing app designs were streamlined, colorful, and intuitive with emphasis on visuals. Notably, mHealth apps designed for childhood asthma were predominantly educational, affirming our unique perspective targeting parent–child shared management responsibility.

### Ideation

**Figure 3:**
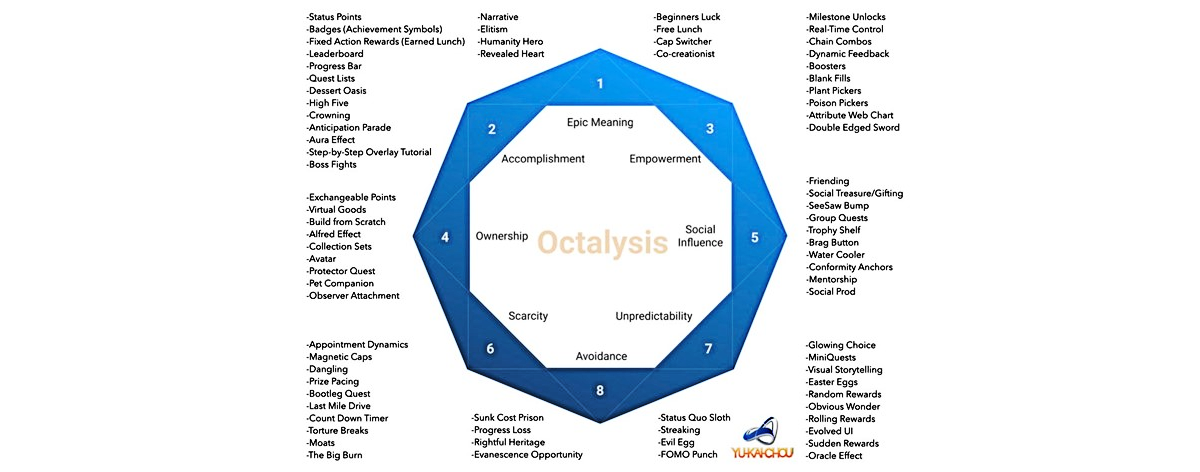
Octalysis Framework for gamification and behavioral design. Reproduced, with permission, from [38]. UI: user interface.

Using the detailed Octalysis Framework (Figure 3), which provides feature examples mapped to each CD, the study team brainstormed and then prioritized various concepts that could be incorporated into our app (Table 2).

**Table 2:** Ideation phase: gamification brainstorming results.

Core drive and concept brainstorm			Priority^a^
**CD 1^b^: Epic meaning (positive motivation)**			
	N/A^c^	N/A	
**CD 2: Development (extrinsic, positive motivation)**			
	Setting and achieving measurable goals (promotes accomplishment)^d^	1	
	Positive reinforcement (congratulations when goal met)^d^	1	
	Leveling up or other reward system^d^	2	
	Progress bar for rewards^d^	2	
	Badges for achievements	2	
	To-do list for app activities^d^	3	
	Step-by-step onboarding^d^	4	
	Hotspots during onboarding^d^	4	
**CD 3: Empowerment (intrinsic, positive motivation)**			
	Choice of a list of recommended goals and option to write own goals (blank fill)^d^	1	
	Real-time feedback during goal progress reports^d^	1	
	Customize app backgrounds or decor with accumulated rewards	4	
**CD 4: Ownership (extrinsic)**			
	Customize profile with avatar^d^	1	
	Virtual pet or mascot^d^	2	
	Accumulated rewards as currency for a virtual “good”	2	
**CD 5: Social influence (intrinsic)**			
	Shared app engagement with parent and child^d^	1	
	Option to share results with health care provider^d^	3	
	Asthma tips from health care professionals (mentorship)	4	
	Chat forum (collaboration)	4	
	Leaderboard to inspire competition	4	
**CD 6: Scarcity (extrinsic, negative motivation)**			
	Progressively more difficult to earn rewards^d^	2	
	Reward options progressively expand with ongoing use^d^	2	
**CD 7: Unpredictability (intrinsic, negative motivation)**			
	Unlocking new rewards^d^	2	
	Streaks	3	
**CD 8: Avoidance (negative motivation)**			
	Surprise rewards	3	

^a^1=highest priority and 4=lowest priority.

^b^CD: core drive.

^c^N/A: not applicable.

^d^Depicts concepts that emerged in the final prototype.

### Design

#### Overview

As depicted in Figure 1, this project entailed 6 cycles of design and evaluation such that insights from evaluation informed future design refinements. The design foci, evaluation methods, and key insights of each cycle are discussed below and in Table 3. Several concepts were never prototyped, including chat forums and leaderboards, due to patient privacy concerns. Expert asthma tips were not prototyped as these are redundant with existing asthma apps. We primarily used medium-fidelity Figma prototyping during design (and evaluation), which entails certain clickable elements within the design. For the last design and evaluation cycle, a high-fidelity Figma prototype was used, which had fully integrated clickable elements such that users could simulate real-world use and navigate as if it were a real app.

**Table 3:** Design foci, evaluation, and key insights by cycle (N=12 dyads).

Cycle	Participants and visit type	Design foci	Evaluation	Key insights
1	n=6 dyads In-person	*Baseline mid-fidelity prototype functionality and layout:* Dashboard: Asthma Symptoms and C-ACT^a^ Goal-setting flow Progress reporting flow	*Concept test:* Dashboard display for asthma symptoms and C-ACT (PP^b^) *Usability test:* Goal-setting flow (TA^c^, SS^d^) Progress report flow (TA, SS)	*Concept test:* Separate symptom and C-ACT graphical displays. Simplify layout. Modify color scheme. *Usability test:* Retain goal choices, improve flow. Response or celebration for achieved goal. Need more child engagement. Good parent–child interaction during use.
2	n=3 dyads In-person	*Mid-fidelity prototype functionality and layout:* Add avatar for user profiles Simplified dashboard Revised goal-setting flow Enhanced progress reporting flow C-ACT flow	*Concept test:* Various avatar options (PP) *Usability test:* Goal-setting flow (TA, SS) Progress report flow (TA, SS) ACT completion and interpretation (TA, SS)	*Concept test:* Preferred animal avatars. Liked ability to customize. *Usability test:* Flows improved and clear. Love celebration response when goal achieved. Color schemes much improved. Add legend for C-ACT interpretation.
3	n=3 dyads Remote	*Mid-fidelity prototype and child engagement strategies:* Introduce animal mascot and reward system concepts Revise C-ACT flow with legend	*Concept test:* Animal mascots (PP) Color themes (PP) Reward systems (TA) *Usability test:* Legend for C-ACT (TA, SS)	*Concept test:* Love the animal mascot and rewards, preferred dog theme. Integrate animal mascot with reward system. *Usability test:* C-ACT legend is clear. Show symptoms first on dashboard. New feature suggestions—medication tracking, data export.
4	n=6 dyads Remote	*Mid-fidelity prototype and animal mascot:* Introduce home spirometer concept Refined dog mascot and reward theme	*Concept test:* Spirometer integration with app (SS) *Usability test:* Dog mascot with bone reward system (TA, SS)	*Concept test:* All participants desire home spirometer integration. *Usability test:* Children love dog mascot that grows with rewards. Easily understood reward system, progress bars.
5	n=5 dyads Remote	*Mid-fidelity prototype and introduce concepts:* Onboarding To-do list New background or accessories Streak Medication tracking Spirometer in dashboard and performance incentive	*Concept test:* Onboarding (PP) To-do list (PP) Background and accessories (TA, SS) Streak (PP, SS) Medication tracking (PP, SS) Spirometer in dashboard (TA, SS)	Dyads preferred sequential onboarding flow with prompts. Scrollable to-do list with “done” checkmarks. Add prompt on dashboard if there are items to do. Children did not care for new backgrounds or accessories. Prefer additional pets/mascots. Children did care for streaks. Dyads prefer calendar plus reminders for medication tracking. Spirometer tracking needs to be simplified with export function. Different spirometer performance incentive (windmill) ok, but could be improved.
6	n=5 dyads Remote	*High-fidelity prototype:* *Introduce:* Animal mascot unlocks “Maintenance phase” *Refine:* Onboarding To-do list Medication tracking Spirometer incentive and tracking	*Concept test (TA, SS):* New animal mascot unlocks (TA, SS) Maintenance phase concept (TA, SS) *Usability test:* Onboarding (TA, SS) To-do list (TA, SS) Medication tracking (TA, SS) Spirometer incentive and tracking (TA, SS)	*Concept test:* Love animal mascot unlocks. Love maintenance phase concept, suggest “mute” for goals, retain everything else. *Usability test:* Refinements were all clear. Keep calendar for medication use and add option for medication reminders. Consider minor edits for consistent language, color scheme.

^a^C-ACT: Childhood Asthma Control Test.

^b^PP: parallel prototyping.

^c^TA: think aloud.

^d^SS: semistructured interview.

#### Cycles 1 and 2

The highest priority items from the ideation phase related to core functions within the app, specifically evaluating goal setting, the interaction between parent and child, and a new priority, an avatar for child users to excite them about ongoing use of the app. These items cross numerous CDs. Based upon these priorities, the designer expanded the original prototype to include positive reinforcement when a dyad reported successful achievement of a goal. An avatar concept was also prototyped to customize user profiles, with various options including animals, robots, dinosaurs, and monsters. These priority items were tested with users in cycles 1 and 2, with refinements made after each cycle based upon user feedback (described in the “Evaluation” section).

#### Cycles 3 and 4

**Figure 4:**
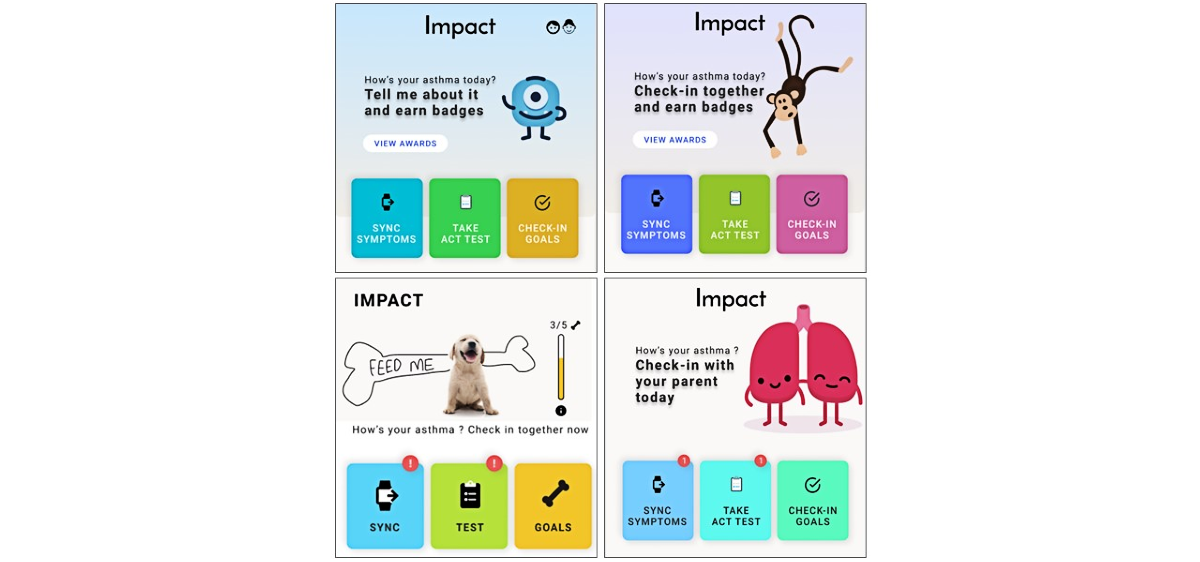
App mascot prototypes.

The next batch of priority concepts related to developing a virtual pet or mascot and reward system for app usage, with the intent to promote and maintain child user engagement. Numerous prototypes were developed with animal or creature mascots, including a monkey, underwater theme, animated lungs, monster, and a dog (Figure 4). Several rewards systems were similarly developed such that users earned rewards for various goal achievements. A variety of progress bars, displays options, and rewards were prototyped around the mascot concept.

#### Cycles 5 and 6

The final design cycles focused on several of the items from Table 2, including 2 versions of a to-do list, onboarding guidance, and several longitudinal app engagement concepts. Onboarding guidance prototypes included carousels of app features, spotlights on app functionality, and options for video tutorials. Longitudinal engagement strategies included expanding the animal mascot concept to provide options for unlocking additional mascots, using accumulated rewards to purchase new app backgrounds or accessories for the animal, and the concept of streaks. We also designed for integration of a home-based spirometer within the app, which emerged from parent participant feedback during prior remote evaluation sessions amidst the COVID-19 pandemic. The study team developed a spirometry tracking graph within the IMPACT app dashboard as well as visual incentives for optimal spirometry performance. Finally, we designed an export feature, which would provide users the option to export app data (C-ACT, spirometry results, etc.) to their health care provider.

### Evaluation

#### Overview

**Figure 5:**
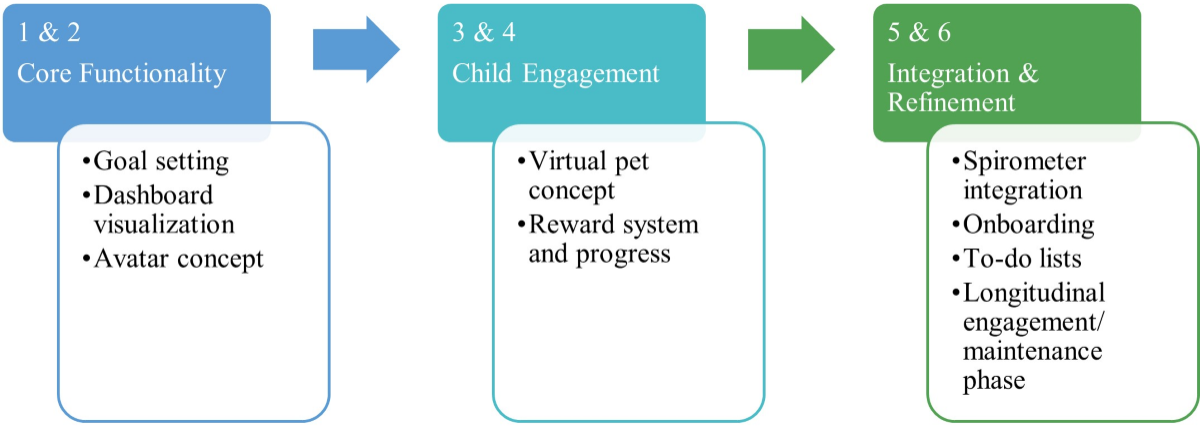
Design and evaluation foci by cycle.

Most evaluation cycles used concept testing, to determine specific concepts to integrate within the app, as well as usability testing, to determine whether users were able to use the implemented concepts as intended. Overall, evaluation cycles initially prioritized core app functionality, then child engagement, and finally integration of all concepts and refinement (Figure 5). Key insights by cycle are presented in Table 3.

#### Cycles 1 and 2

To test the core app functionality, cycles 1 and 2 prioritized evaluating the original prototype dashboard and goal-setting flows. The dashboard, or home screen, depicts graphical representations of the child’s asthma symptoms and a weekly C-ACT. Concept testing was used to determine parent and child preferences for dashboard layout and color schemes.

I would totally have nothing on here except the symptom box and move everything down to the navigation bar...so you are only focusing on which is important, which is symptoms.

P3

We also concept tested avatar options with child participants, with animal avatars emerging as preferred.

Usability testing was used to test the goal setting, progress report, and ACT flows. The goal-setting flow was very clear, with one parent commenting,

Oh boy, I love these goals!

P2

During the semistructured interview, another parent asked what would happen after dyads completed the intervention. This parent suggested that the team consider an additional app phase that did not focus on changing shared management behaviors, but rather on maintaining them along with ongoing asthma symptom and control tracking (later addressed in design cycle 6). The remaining flows tested well with minor refinements recommended.

#### Cycles 3 and 4

Cycles 3 and 4 focused on evaluating the animal mascot and reward system child engagement strategies. A series of parallel prototyping concept tests revealed that dyads strongly preferred a dog mascot and that the reward system be cohesive with the mascot. Ultimately, a dog bone reward scheme was selected. During the interview, one child suggested we build on the dog mascot concept:

You can feed the animals to make them bigger and better...and they can get different colors.

C3, 11 years, during cycle 3

This suggestion was integrated into cycle 4, with the dog mascot initially displayed as a puppy that progressively grows through reward achievements. Usability testing revealed the growing puppy mascot was unanimously favored.

Amidst the COVID-19 pandemic, many parents with children with asthma expressed concern about decreased access to their health care provider. A specific concern was restrictions placed on spirometry, which is an aerosolizing procedure. In light of these parent-identified concerns, we introduced an additional concept for testing, home spirometry. One parent commented,

Yes, that would be extremely useful. That would give us the information to decide whether to go to the doctors or emergency.

P1

Another shared,

I think this would be great! It also generates more data to give to the doctor...He [the child] might be fine when he goes to see the doctor once a year, but the rest of the year he wheezes, and I worry.

P2

Finally, interviews revealed that dyads would also like to see the app incorporate medication tracking and reminders.

Medication reminders. We need a reminder...that’s what we need more than anything.

P1

#### Cycles 5 and 6

Cycle 5 was entirely focused on concept testing various options for onboarding, app tasks, streaks, medication tracking, and spirometry integration. Dyads were very decisive on preferred formats. Child participants clearly did not care for the streak concept,

Streaks just feel more like work.

C4, 10 years

Children also preferred that we expand the mascots to allow for additional dog “unlocks” within the reward system as opposed to earning accessories or app background changes. The introduction of medication reminders and tracking as well as spirometry were both unanimously favored.

Cycle 6 concept testing evaluated the maintenance phase concept to follow intervention completion, which originated from a parent participant, whereby the goal setting was muted, and dyads may continue to use the other app features long term. Dyads loved this addition as it would enable ongoing app use even after the intervention was complete. High-fidelity usability testing revealed only minor refinements, indicating readiness to move to implementation.

### Implementation

Once the high-fidelity prototype was finalized, the study team worked alongside the engineering team to construct user stories that specify feature requirements within the app. User stories are written from the perspective of the user, such as “as a user, I want to be able to track my asthma control within the app” [48]. Screenshots, Figma prototype links, and descriptions of the related prototype features often accompanied the user stories. Globally, user stories help provide the “why” for software developers alongside the prototype [49]. Practically, they constitute a step-by-step guide for development of a product and subsequent internal testing to ensure the developed app functions as intended [48-50]. Developer effort and timelines are also estimated based upon user stories. Given that the development and study teams co-constructed the user stories, any questions or clarifications related to the proposed design were addressed collaboratively. In total, these user stories constitute the design specifications for the engineering team.

## Discussion

### Principal Findings

The purpose of this study was to use HCD to refine the original parent–child shared asthma management IMPACT app and to incorporate longitudinal app engagement strategies. The study team successfully refined the app, incorporated longitudinal engagement strategies, and added dyad-prioritized new features. Final testing indicated that parent–child dyads found the refined IMPACT prototype addressed their prevailing asthma needs and priorities in an engaging, easy-to-use app.

Partnering with our end users ensured that our final design met the needs and priorities of children with asthma and their parents. Evidence has shown that such participatory design practices increase the likelihood of intervention uptake and efficacy [51,52]. Our participants were very clear that they did not desire another educational intervention, but rather a system to address challenges in monitoring symptoms and transitioning asthma responsibility to the child in a safe manner. Parent participants were especially enthusiastic about the shared management goals, which break down key asthma management tasks while facilitating parent–child shared monitoring and management. The incorporation of spirometry was also in direct response to a serious parental concern about barriers to health care access amidst the COVID-19 pandemic. Our study also serves as an example for integrating other mHealth best practices, including theoretically informed intervention techniques, clinical guidelines, and validated assessment tools [53-55].

Drawing upon the Octalysis Framework, our final design successfully integrated concepts from 6 of the 8 gamification CDs, excluding CD 1 (epic meaning) and CD 8 (loss and avoidance). Epic meaning, or being part of something bigger than oneself, does not align well with an mHealth app, which is specifically designed to support an individual’s health. We concept tested a CD 8 concept, streaks, though none of our participants recommended retaining the concept. The final IMPACT design included more extrinsic than intrinsic motivations, though both are accounted for in the design. This is not unexpected as extrinsic motivations, such as tangible rewards, are more straightforward for children and frequently used in child-facing gamified systems [34,56-58]. Similarly, some intrinsic motivations, such as social sharing, are inappropriate for mHealth apps due to patient confidentiality concerns. However, an ideal gamified system does not need to integrate all CDs, but rather ensure each of the 4 motivation dimensions are accounted for in the design [36].

Despite the surge in mHealth interventions, to our knowledge, none have been designed to promote parent–child shared asthma management. Just as children need to learn self-care practices in a stepwise fashion (ie, feeding, dressing), so too do they need to learn self-management in a similar progression. Unfortunately, evidence shows that youth often abruptly assume complete management of their asthma during adolescence, often resulting in worsened health status and poor health outcomes [11]. Despite this evidence, guidance facilitating parent–child shared asthma management is lacking in the literature and existing mHealth apps [11]. Interventions specifically designed to facilitate safe and intentional parent–child shared management through concrete, task-based goal setting represent a novel approach to teaching children essential asthma management skills while still under the supervision of their parent. Such innovations hold the promise of improving a child’s health in the present as well as building lifelong self-management skills.

To our knowledge, there are few, if any, studies describing the iterative design of a dyadic health management app [59]. Our study represents an exemplar for integrating mHealth best practices, particularly behavior change, personalized self-monitoring, and medical guidelines [27,30], while concurrently accounting for a dyad’s unique circumstances, family needs and priorities, and social environment [45,59]. As equal participants, parent and child feedback was carefully considered and incorporated into the app to ensure that the needs of both types of users are accounted for. While we anticipated parental hesitation to allow their child to assume asthma responsibility, we actually found that most were relieved and excited to be developing an app that would meet their needs. Multiple rounds of design and evaluation were necessary to successfully incorporate these preferences within the app; dyads often would resolve disagreements about app designs without study team facilitation. The result was an mHealth solution that represents not only a solution to dyad-identified needs and priorities, but also one that models a paradigm shift from personal to family informatics [59]. Such solutions hold tremendous promise in supporting families in navigating parent–child shared management of health.

### Limitations

While this study has many strengths, there are important limitations that warrant consideration. The convenience sample of parent–child dyads was small and somewhat homogenous (primarily male children and female parents), which limits generalizability of study findings. The sample was recruited from the principal investigator’s research database, which is not representative of all school-age children and their parents and did not screen for other comorbidities. It is also possible that study dyads experienced social desirability bias. Finally, our recruitment was confined to one geographic area, again limiting potential generalizability of our findings.

### Conclusions

The final IMPACT app is a theoretically derived, tailored parent–child shared asthma management intervention and monitoring system. IMPACT was iteratively co-designed by our interdisciplinary study team as well as end users to ensure that the app meets the needs and priorities of children with asthma and their parents. The final IMPACT prototype is presently being fully developed for an anticipated 8-week pilot RCT in which we will test the feasibility, acceptability, and preliminary efficacy of the app.

## Abbreviations

AM: affinity mapping
C-ACT: Childhood Asthma Control Test
CD: core drive
HCD: human-centered design
IMPACT: Improving Asthma Care Together
mHealth: mobile health
PP: parallel prototyping
SS: semistructured interview
TA: think aloud
UX: user experience
